# Varying image assessment of pecking injuries in Turkeys while performing repetitions

**DOI:** 10.1007/s11259-025-10833-6

**Published:** 2025-08-08

**Authors:** Nina Volkmann, Lars Schmarje, Reinhard Koch, Nicole Kemper

**Affiliations:** 1 Institute for Animal Hygiene, Animal Welfare and Farm Animal Behavior (ITTN), University of Veterinary Medicine Hannover, Foundation, Hannover, Germany; 2 Department of Computer Science, Faculty of Engineering, Christian-Albrechts-University Kiel, Kiel, Germany

**Keywords:** Image assessment, Reliability, Observer bias, Repetitions, Pecking injuries

## Abstract

This study investigated variations in assessing potential pecking injuries in turkey hens when annotating image excerpts. Three observers (OBS1, OBS2, OBS3) with different levels of previous knowledge - one with experience in pecking injuries in turkeys and two computer science students - rated a total of 24,912 image excerpts. The image excerpts were evaluated in work packages (2,076 images each) and were classified by the observers as either head injury (HI), skin injury in the feathered area of the body (SI), or no injury (NI). Two observers evaluated three packages (OBS1, OBS2: 6,228 image excerpts each) and OBS3 annnotated six work packages (12,456 excerpts). The percentage of the classifications in the chronological sequence of the observations was analyzed. Inexperienced observers (OBS2 and OBS3) both classified an average of 13% of the shown images as HI, 70% as SI, and 17% as NI. On average, OBS1 classified 12% of the images as HI, 60% as SI, and 28% as NI. Throughout the study, all observers classified more recordings into the NI class. Particularly, OBS1 with the most experience in evaluating pecking injuries showed a different assessment by rating more images (plus 5%) as showing NI over time (OBS2: plus 0.7%; OBS3: plus 2.2%). This result raises questions about whether divergent assessments always occur in repeated judgments and how this effect can be avoided.

## Introduction

Consecutive assessments of animals are common in scientific research. For example, previous studies evaluated the plumage in chickens (Spindler et al. 2020), footpads in turkeys (Stracke et al. 2020), and tail lesions in pigs (Brünger et al. 2019) using a scoring system assessed by human observers. However, it is well-known that these human assessments can be subjective and inaccurate due to a lack of objectivity. As Tuyttens et al. (2014) pointed out, researchers are prone to strong expectations about study results, and observers are influenced by assumptions about what is possible and what is not. This is a pitfall of the human brain, leading to favoring information that confirms previous assumptions (Nickerson 1998). Thus, recorded data and observations can be biased due to the expectations of the human observer. Observer-blinded studies are intended to reduce conscious and unconscious bias, and ensure that observers collecting data are unaware of which animals have been treated or affected by a disease, for example (Tuyttens et al. 2014).

Statistical methods to verify the reliability of human observers include inter- and intra-observer tests and examining test-retest agreement. Inter-observer reliability can be checked by training multiple humans to rate the same individual or image independently (e.g. Blömke et al. 2020). This way, the reliability between the observers can be examined in advance by calculation indices like Pearson’s and Spearman’s correlation, Krippendorff’s alpha or Cohen’s kappa. In order to make such comparisons between observers, the data is assigned to meaningful units such as scores or classes which then can serve as a so-called gold standard (Hooge et al. 2018). To characterize the behavioural pattern of animals, an ethogram is generated, which is a comprehensive description of the behavioural units and should be the starting point of every ethological study (Lehner 1996). However, many studies do not have a gold standard for comparison or collect quantitative data and instead perform a subjective visual assessment to determine and validate quantitative characteristics. Thus, without clear definitions or specifications, measuring whose evaluation is ‘more correct’ or whether an observer has rated correctly is difficult.

An intra-observer reliability test in research studies is used to verify if a human observer achieves the same result when measuring the same observation at different moments (Bokkers et al. 2012). Test-retest reliability is used to evaluate the investigation method or examine the agreement between results on the same test conducted at two different times. This type of test refers to the chance that the same outcome will be observed if the trial is repeated (Temple et al. 2013).

To improve reliability and agreement of observers training is essential (Kaufman and Rosenthal 2009). Training on the scientific question in advance is a prerequisite for observer agreement and enables its improvement. However, Schlageter-Tello et al. (2015) showed that experienced and inexperienced observers were unable to improve reliability and agreement after a short training and evaluation of the locomotion of about 600 cows. Their results suggest that, in addition to training, the experience of the observer is also an important aspect (Schlageter-Tello et al. 2015). In addition, Kazdin (1977) stated that even after training and with experience, the tendency of observers to apply the definitions of a measurement changes over time.

The present study was conducted as part of a main research project on different annotation types (Schmarje et al. 2022) and aimed to examine retrospectively whether different observers, when assessing potential pecking injuries in turkeys, tended to change their classification when repeatedly viewing the same images of injured and uninjured animals. The aim of the study was defined after the implementation of the main study, and the data was evaluated accordingly.

## Materials and methods

**Fig. 1:**
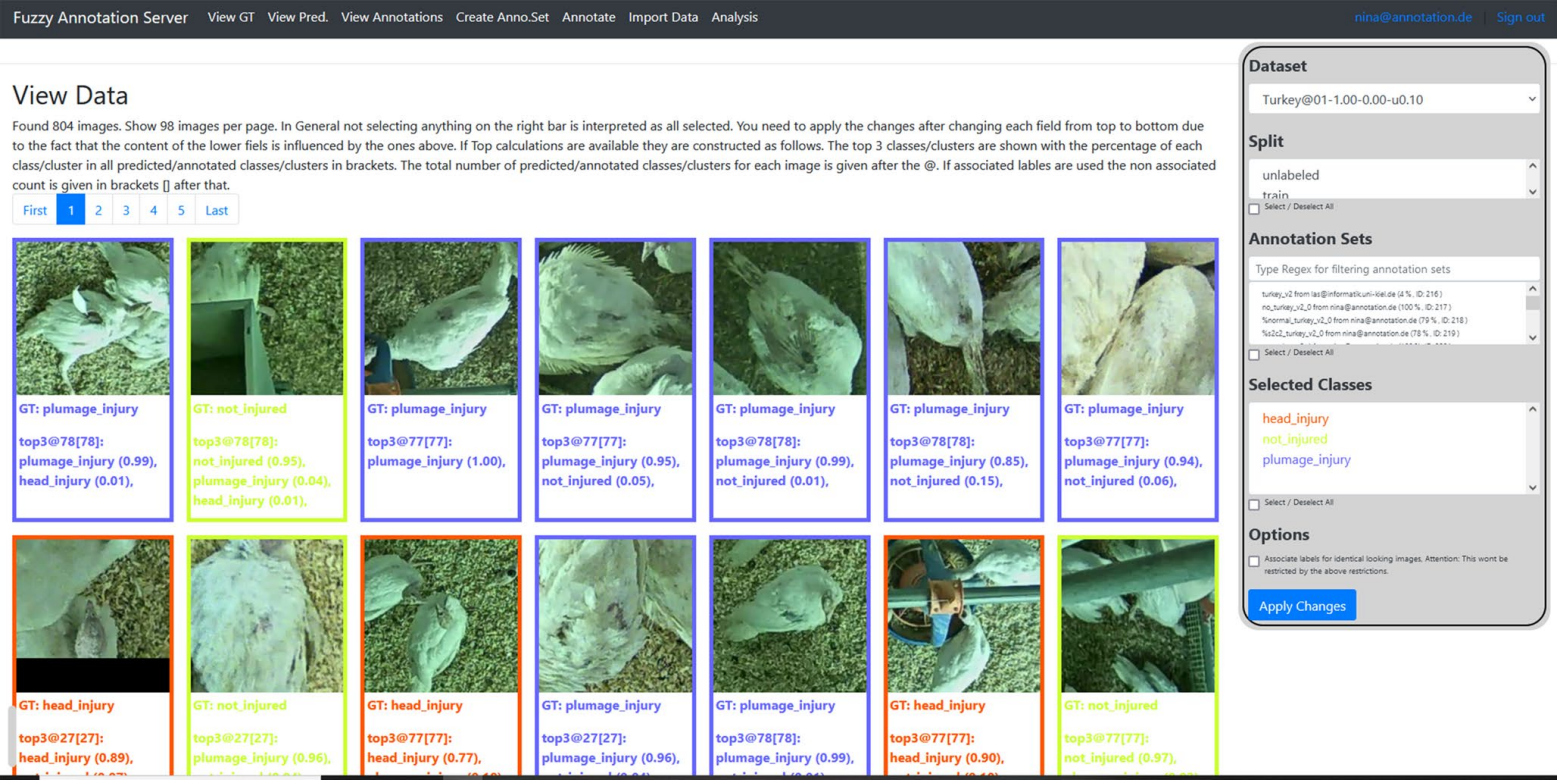
Screenshot of the developed web server showing example image excerpts for annotation assessment

For investigations, videos from a previous study on pecking injuries in turkeys (Volkmann et al. 2021) were used which were recorded on a German research farm with female turkeys using three top-view cameras (AXIS M1125-E IP camera, Axis Communications AB, Lund, Sweden) installed approximately 3 m above the floor. For the further processing, video recordings were divided into individual frames and cut into image excerpts with 224 × 224 pixels in size showing turkey hen(s) or parts of the animal (with or without one existing injury) or objects in the stable such as a pick block. These image excerpts were made available on a web server in a zoom view for annotation (Fig. 1) and were provided as a work package (WP) with image excerpts, whereby each package in turn consisted of three annotation sets (Set 1: 804 images, set 2: 636 images, set 3: 636 image excerpts). The fact that a work package consisted of three annotation sets resulted from the main study (Schmarje et al. 2022), which investigated the impact of (semi-)supervised methods on data quality in image classification.

The work included the annotations of three observers, one of whom (researcher in the field of animal welfare) had prior experience with pecking injuries in turkeys (OBS1), while the other two were computer science students (OBS2 and OBS3). Before performing their individual annotations, the observers completed joint online trainings, which were led by the experienced observer. In this previous trainings, they evaluated randomly selected image excerpts (*n* = 4,152) together and discussed their results.

Afterwards, the three observers evaluated the work packages, which consisted of 2,076 image excerpts each, on their own and thus without further consultations. Whitin one package the observers assessed the image excerpt in the three annotation sets 1–3. The process of evaluating a work package was then repeated by the observers at a time interval of their choice (all packages were evaluated by the respective observers within one month).

During the annotation process, the observers had to assign each image excerpt to one of three different classes: ‘head injury’ (HI), ‘skin injury in the feathered area of the body’ (SI), or ‘no injury’ (NI). OBS1 and OBS2 each evaluated three work packages (in total 6,228 image excerpts each observer), while OBS3 evaluated six work packages (in total 12,456 image excerpts). Since the image excerpts in each work package were evaluated one after the other, the three observers classified the same images in the same order.

The results of their assessments were recorded, and after the main study (Schmarje et al. 2022) was completed, the corresponding hypothesis for the present study was set, which was finally evaluated. Thus, in this ‘secondary’ study it was retrospectively not possible to calculate an observer agreement based on individual image excerpts.

For statistical analyses, the classes containing ‘head injury’ (HI) and ‘skin injury’ (SI) were summarized to one named ‘injured’. The results on the frequency to which the images were assigned to the class ‘no injury’ (NI) and ‘injured’ respectively were analyzed using the SAS software (Version 9.4, Statistical Analysis Institute, Cary, NC, USA). To show a temporal effect during annotation process, a Fisher’s exact test was conducted investigating the association between the mean frequencies of the selected annotation class (‘injured’ and NI) and the work package (of the individual observer). P values of < 0.05 were considered statistically significant.

## Results

The results of the individual percentage classifications of the three observers within each work package and annotation set are shown in Table 1.

**Table 1:** Percentage rating of the individual observers (OBS1, OBS2, OBS3) of the image excerpts

			OBS1			OBS2			OBS3		
WP	S	n	SI	HI	NI	SI	HI	NI	SI	HI	NI
1	1	804	68.7	11.3	20.0	78.1	8.8	13.1	75.4	11.8	12.8
	2	636	61.5	12.3	26.3	67.1	13.8	19.0	68.9	13.2	17.9
	3	636	57.6	12.4	30.3	65.3	16.7	18.1	68.1	15.1	16.8
2	1	804	61.6	11.1	27.4	76.6	11.1	12.3	76.1	11.9	11.9
	2	636	58.7	13.4	28.0	69.5	11.3	19.2	70.4	12.9	16.7
	3	636	57.3	12.6	30.2	63.8	15.9	20.3	67.3	14.9	17.8
3	1	804	59.6	10.6	29.9	76.2	10.8	12.9	73.0	12.7	14.3
	2	636	55.2	12.3	32.6	65.6	16.0	18.4	73.0	12.9	14.2
	3	636	56.9	13.1	30.0	65.7	13.4	20.9	68.2	13.5	18.2
4	1	804							71.6	11.6	16.8
	2	636							67.0	13.1	20.0
	3	636							66.4	12.7	20.9
5	1	804							70.5	12.2	17.3
	2	636							67.0	12.9	20.1
	3	636							65.4	14.2	20.4
6	1	804							72.8	11.9	15.3
	2	636							67.6	13.1	19.3
	3	636							66.5	13.8	19.7

*WP* work package, *S* number of the annotation set, *n* number of image excerpts, *SI* skin injury in the feathered area of the body, *HI* head injury, *NI* no injury

Inexperienced observers (OBS2 and OBS3) classified an average of 13% of the shown images as HI, 70% as PI, and 17% as NI. On average, OBS1 classified 12% of the images as HI, 60% as PI, and 28% as NI, thus evaluating more image excerpts with NI than the two inexperienced observers.

**Fig. 2:**
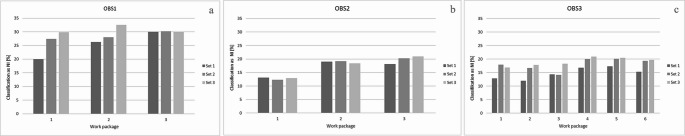
**a**–**c** Percentage of image excerpts which were classified by the observers (OBS1, OBS2, OBS3) as ‘no injury’ (NI). The annotation sets (Set 1–3) of the work packages were assessed one after the other

For all three observers, it was observed that the proportion of images assessed as NI increased over time as the images were repeatedly evaluated (Fig. 2a-c). Consequently, the ratio of image excerpts potentially showing an injury decreased. This effect was most pronounced in OBS1, who additionally rated more than 5% of the images in the third work package as NI (WP1: 25.5% on average; WP3: 30.8% on average) (Fig. 1a). However, analyzing the mean frequencies of the annotated injury classes (‘injured’ vs. ‘no injury’), Fisher’s exact test revealed no assosiation between these classification and the work packages (all *p* > 0.05).

## Discussion

The purpose of this study was to investigate different classifications of pecking injuries in turkeys repeatedly perfomed by observers with various prior experience.

The study results showed that the repeated performance of annotation assessments, such as the classification of pecking injuries in turkeys, can lead to variable judgments. In a study by Thomsen et al. (2008), a locomotion scoring experiment for dairy cows was repeated after one week and an additional training was carried out. At the second run, the observers showed slightly decreased intra-observer agreement by evaluating cows’ walk simultaneously in the barn. Thomson et al. (2008) assumed, that the short time between both scorings decreased the possibility that cows’ true lameness status could change from the first to the second run, and they stated that observer training seemed to have had only minor effects on agreement.

In the present study, modifying the assessment regarding potential pecking injuries may have led to a ‘deadening’ effect among the observers, as the number of animals classified as not injured decreased. It is also possible that the observers improved their view on the image excerpts over time and thus this development can be interpreted as positive learning effect. In a study on reliability and accuracy of detecting keel bone damage in hens an improvement in the palpation assessment was observed through repetition, which was recognized by comparing the results with those from radiography and sonography (Tracy et al. 2019). Furthermore, Tracy et al. (2019) observed a very large variation between the palpation results of the differently experienced observers. However, in the present study, the results of the one experienced observer (OBS1) can probably not be explained by a learning effect. Here, the experienced observer was particularly practiced and trained in assessing pecking injuries on images and yet the effect evaluating more excerpts with NI was more pronounced in OBS1 than in the computer science students (OBS2 and OBS3). Thus, it is conceivable that inexperienced, non-expert observers may be able to judge more objectively and may not hinder high-quality results in such studies but rather improve them. Of course, this depends on the complexity of the evaluation procedure. In the present study, less prior knowledge was needed than in situations where an observer is asked to judge, for example, the gait pattern means of a multistage locomotion score (Winckler and Willen 2001) or if a state of health is to be recorded based on medical expertise (Baadsgaard and Jørgensen 2003). Nevertheless, a ‘deadening’ effect is only suspected due to the altered annotations of one single trained observer. In order to verify this assumption, repeated observations should be carried out by several (experienced) participants.

When presenting the results obtained regarding the repeated judgment, it is essential to remember that there was no gold standard or ethogram included in this study. Therefore, it cannot be stated which assessment was correct and whether the changed assessment improved the results or not. However, it can be assumed that computer science students, lacking prior knowledge of pecking injuries in turkeys, resulted in less bias due to expectations in evaluating the images. Such assessment bias is likely when the observer has strong preconceptions or a vested interest in the outcome (Tuyttens et al. 2014).

One limitation of this study is the lack of an intra- and inter-reliability test between the observers. As these evaluations were only conducted after the main study (Schmarje et al. 2022) was completed, it was not possible to conduct an observer comparison retrospectively. Therefore, the results of this study should rather encourage a closer look at the possible temporal changes in the evaluation of image assessments. Nevertheless, there is no question that intra- and inter-reliability tests should be carried out in further studies. Furthermore, image assessments should be repeated, and observation changes should be considered. In an article by Risinger et al. (2002) regarding observer effects due to expectations, an example is given where the observer sees something different in a drawing depending on which similar images he/she has previously viewed. Thus, when evaluating images, the evaluation process should be performed on the same photos in a different order to avoid or detect some kind of ‘deadening’ - since the evaluation of an animal that is only slightly sick or has only a small injury can be different if one has seen many severely sick animals or severe injuries beforehand.

In summary, it is noted that the ‘deadening’ effect assumed in this brief report should be tested in further research using a different study design. Nevertheless, it is expected that, analogous to the assumptions regarding ‘deadening’ in this study, other ratings/classifications are not free from effects such as habituation, time, or tiredness. Further studies should investigate to what extent inexperienced, non-specialist observers can judge more objectively or from which number of images such as ‘deadening’ can be observed.

## Data Availability

Data available on request from the authors.

## References

[CR1] Baadsgaard NP, Jørgensen E (2003) A bayesian approach to the accuracy of clinical observations. Prev Vet Med 59(4):189–206. 10.1016/S0167-5877(03)00100-4 12835004 10.1016/s0167-5877(03)00100-4

[CR2] Blömke L, Volkmann N, Kemper N (2020) Evaluation of an automated assessment system for ear and tail lesions as animal welfare indicators in pigs at slaughter. Meat Sci 159:107934. 10.1016/j.meatsci.2019.107934 31493738 10.1016/j.meatsci.2019.107934

[CR3] Bokkers EAM, de Vries M, Antonissen I, de Boer IJM (2012) Inter- and intra-observer reliability of experienced and inexperienced observers for the qualitative behaviour assessment in dairy cattle. Anim Welf 21:307–318. 10.7120/09627286.21.3.307

[CR4] Brünger J, Dippel S, Koch R, Veit C (2019) Tailception’: using neural networks for assessing tail lesions on pictures of pig carcasses. Animal 13(5):1030–1036. 10.1017/S1751731118003038 30428955 10.1017/S1751731118003038

[CR5] Hooge I, Niehorster DC, Nyström M, Andersson R, Hessels RS (2018) Is human classification by experienced untrained observers a gold standard in fixation detection? Behav Res Methods 50(5):1864–1881. 10.3758/s13428-017-0955-x 29052166 10.3758/s13428-017-0955-x PMC7875941

[CR6] Kaufman AB, Rosenthal R (2009) Can you believe my eyes? The importance of interobserver reliability statistics in observations of animal behaviour. Anim Behav 78(6):1487–1491. 10.1016/j.anbehav.2009.09.014

[CR7] Kazdin AE (1977) Artifact, bias, and complexity of assessment: the ABCs of reliability. J Appl Behav Anal 10:141–150. 10.1901/jaba.1977.10-141 16795543 10.1901/jaba.1977.10-141 PMC1311161

[CR8] Lehner PN (1996) Handbook of ethological methods, 2nd edn. Cambridge University Press, Cambridge, p 665

[CR9] Nickerson R (1998) Confirmation bias: A ubiquitous phenomenon in many guises. Rev Gen Psychol 2:175–220. 10.1037/1089-2680.2.2.175

[CR10] Risinger DM, Saks MJ, Thompson WC, Rosenthal R (2002) The daubert/kumho implications of observer effects in forensic science: hidden problems of expectation and suggestion. Cal L Rev 90:1. 10.2139/ssrn.301408

[CR11] Schlageter-Tello A, Bokkers EA, Groot Koerkamp PW, Van Hertem T, Viazzi S, Romanini CEB, Halachmi I, Bahr C, Berckmans D, Lokhorst K (2015) Relation between observed locomotion traits and locomotion score in dairy cows. J Dairy Sci 98:8623–8633. 10.3168/jds.2014-9059 26387018 10.3168/jds.2014-9059

[CR12] Schmarje L, Grossmann V, Zelenka C, Dippel S, Kiko R, Oszust M, Pastell M, Stracke J, Valros A, Volkmann N, Koch R (2022) Is one annotation enough? A data-centric image classification benchmark for noisy and ambiguous label estimation. 36th Conference on Neural Information Processing Systems (NeurIPS 2022), 13 Oct 2022

[CR13] Spindler B, Weseloh T, Eßer C, Freytag S, Klambeck L, Kemper N, Andersson R (2020) The effects of UV-A light provided in addition to standard lighting on plumage condition in laying hens. Animals 10:1106. 10.3390/ani10061106 32604949 10.3390/ani10061106 PMC7341212

[CR14] Stracke J, Klotz D, Wohlsein P, Döhring S, Volkmann N, Kemper N, Spindler B (2020) Scratch the surface: histopathology of footpad dermatitis in Turkeys (Meleagris gallopavo). Anim Welf 29:19–432. 10.7120/09627286.29.4.41932226239

[CR15] Temple D, Manteca X, Dalmau A, Velarde A (2013) Assessment of test-retest reliability of animal-based measures on growing pig farms. Livest Sci 151:35–45. 10.1016/j.livsci.2012.10.012

[CR16] Thomsen PT, Munksgaard L, Tøgersen FA (2008) Evaluation of a lameness scoring system for dairy cows. J Dairy Sci 91:119–126. 10.3168/jds.2007-0496 18096932 10.3168/jds.2007-0496

[CR17] Tracy LM, Temple SM, Bennett DC, Sprayberry KA, Makagon MM, Blatchford RA (2019) The reliability and accuracy of palpation, radiography, and sonography for the detection of keel bone damage. Animals (Basel) 9(11):894. 10.3390/ani9110894 10.3390/ani9110894 PMC6912489 31683826

[CR18] Tuyttens FAM, de Graaf S, Heerkens JLT, Jacobs L, Nalon E, Ott S, Stadig L, Van Laer E, Ampe B (2014) Observer bias in animal behaviour research: can we believe what we score, if we score what we believe? Anim Behav 90:273–280. 10.1016/j.anbehav.2014.02.007

[CR19] Volkmann N, Brünger J, Stracke J, Zelenka C, Koch R, Kemper N, Spindler B (2021) Learn to train: improving training data for a neural network to detect pecking injuries in Turkeys. Anim 11. 10.3390/ani11092655 10.3390/ani11092655 PMC8469856 34573621

[CR20] Winckler C, Willen S (2001) The reliability and repeatability of a lameness scoring system for use as an indicator of welfare in dairy cattle. Acta Agri Scand (Suppl 30:103–107. 10.1080/090647001316923162

